# To a Correspondent

**Published:** 1886-04

**Authors:** 


### TO A CORRESPONDENT.

"Scoticus," sends us a long account of his symptoms from Winnipeg, Manitoba, with a request that we answer through the Journal. We might take up our whole paper with such epistles, every month, and have enough for many numbers ahead. He is troubled with "depression of spirits, and physical and mental languor, combined with a restless and ambitious temperament, which has made life not by any means a happy dream." Now, without quoting further from his list of diseases and ailments, we will say at once that he makes out a very conflicted case, one no good physician would undertake without a thorough personal examination. This same remark will apply to many such letters—more than we have time or ability to answer. There is, in many of the cases, some governing rule that keeps them in employments that make their lives a burden, when, if they were to quit their indoor habit of business, and go to work at some outdoor employment, they would be healthy, hearty, and good natured. Persons that have never tried it can hardly conceive the difference between the noble employments in natures fields, and the pent-up business of city life.

If he thinks it impracticable to give up his present employment, and it is always a choice between the pleasures of health and the pleasure of making money, let him get a health jolting chair, (see adv. in Health Journal,) and try that.

We have no idea that there is any necessity for a son of Old Scotia being melancholy and despondent about himself, over the belief, that he cannot recover his health. Don't give way to melancholy, but get a Volume of Bobby Burns and read whenever you feel it coming, "by the ould church yard."

**Dr. Borck,** of St. Louis, says that asphaltum varnish is the best disinfectant he knows of; it will destroy all germs at once, and no household insects will approach an article of furniture whose interior has been painted with it.

